# Assessment of workplace safety climate among power sector employees: A comparative study of cross-culture employer in Pakistan

**DOI:** 10.1371/journal.pone.0272976

**Published:** 2022-08-15

**Authors:** Anum Arooj, Muzaffar Majid, Asifa Alam, Mian Farooq Bilal

**Affiliations:** 1 College of Earth and Environmental Sciences, University of the Punjab, Lahore, Pakistan; 2 Pakistan Institute of Quality Control, The Superior College Lahore, Lahore, Pakistan; American University of Sharjah, UNITED ARAB EMIRATES

## Abstract

Pakistan’s power sector has undergone extensive reforms to improve its technical and monetary performance over the last two decades. However, despite its fast-growing and hazardous nature, safety research remains limited in this context. This study aims to address this gap by assessing the level of safety climate in the power sector and comparing the safety climate in plants operated by multinational companies (MNCs) and local companies (LCs).To this end, five power plants operating in the southern part of the Punjab region (in Pakistan) were included in this study. The Nordic Occupational Safety Climate Questionnaire (NOSACQ-50), an analytical tool comprising of 50 items across seven dimensions, was used to determine the level of safety climate. An independent *T-Test* was then applied to compare the means in two different setups to draw a conclusion about overall safety climate differences. In MNCs, overall management/leadership perception improved; however, workers in both setups responded similarly in many cases. The lowest observed score in both setups was related to worker’s prioritization of safety and risk non-acceptance. The study highlights the importance of a company’s policies, procedures, and leadership commitments in creating a stronger safety climate by instilling trust in workers. The study further demonstrates that cross-cultural and strong policies devised by multinational companies help to improve the overall safety climate andconcludes that promoting an efficient and positive safety climate in the power sector is a long journey and that can only be achieved if all workers and leaders take on an active role.

## 1.0 Introduction

Occupational health and safety have become subfields of public health research, policy, and practice aimed at improving workplace health and safety standards [1]. Despite the significance of occupational health and safety (OHS) in all spheres of life, it is yet to garner sufficient research attention, particularly in less developed countries. However, some researchers haveemphasized the importance of OHS, as they consider it a social pillar of sustainable development that reduces occupational accidents and illnesses [2]. The demand for industrialization and energy consumption increased the power industry became significant in Pakistan’s economy. The role of OHS is critical in the power sector as this division is a hazardous working environment. According to the Occupational Safety and Health Administration (OSHA) [3], more than 110,000 power line workers engaged in building or repairing power transmission and distribution systems face a wide range of severe and possibly fatal injuries such as falls from heights, electrocutions, and injuries from falling object. Additionally, electrical shock burn injuries (burns), cuts and lacerations, over-exertion, sprains, strains, and bruises are all common causes of non-fatal injuries. The lack of implementation of OHS practices can have severe consequences at thepersonal and organizational level. For instance, according to recent data from the US Bureau of Labour Statistics, 739 workers died as a result of exposure to electricity between 2012 and 2016 [4]. Furthermore, estimates of the direct and indirect costs of workplace injuries and illnesses are as high as $ 250 billion per year [5]. A lack of safety culture and infrastructure, such as management systems, can also result in more fatal accidents.

The Nordic Occupational Safety Climate Questionnaire (NOSACQ-50) developers define safety climate (SC) as "workgroup members’ shared observations about management and worker safety-related guidelines, procedures, and attitudes" [6]. The safety climate theory is based on social psychology, which recognizes that actions result from interactions between a person and their psychological environment [7]. The current study will add to the existing literature on public health within various organizations, especially the power sector. The results of this study will be helpful for department heads, including top leadership such as directors, general managers, and middle management including departmental heads and managers, to take corrective and preventive actions. The assessment of safety climate (SC) is considered a powerful research strategy, as it is a means of gathering information about safety issues before worker accidents occurs [2]. It has many scientific and managerial benefits, including significant impacts on behaviours and attitudes linked to individual or organisational safety [8]. Additionally, it is a vital tool that enables companies to use rubric-based descriptors to evaluate their safety climate maturity in a reliable way [9], thereby improving safety culture, employee quality of life, and accident prevention [10]. The importance of SC was emphasized by Makki & Mosly [11] who argued that it can assist industry owners and contractors by providing details related to attitudes and perceptions that can enhance safety performance. In terms of managerial benefits, the excellence of the safety climate influences three areas: management, site, and enterprise. It is challenging to manage all areas the corporate level owing to the limited managerial resources available. Therefore, developing a strategy that gradually improves the safety climate is necessary. The proportionate importance of each safety climate evaluation factor must be determined by the company to improve it [8].

Previous studies have focused on industries such as aviation, construction [12, 13], transportation, manufacturing plants, and oil and gas [14], and the majority of safety climate studies have been conducted in Western countries, specifically Australia, the United Kingdom, the United States, and Canada [12]. In contrast, this research focusses on the power sector, in the context of Pakistan, a developing country. As such, this researcher effectively opens up new avenues for gaining a better grasp on the safety climate [15]. Thus far, the power sector has been neglected in safety climate research because of a lack of catastrophic accidents and safety issues.

Since 1994, Pakistan’s power sector has undergone extensive reforms that have improved the sector’s technical and monetary performance [16], and several improvements, such as structural modifications, institutional development, and policy advances, have been introduced over the previous two-decades in an attempt to build up a sustainable power industry [17]. According to recent statistics, an average of 164 fatal occurrences have been reported over the last ten years in Pakistan’s electricity sector [17]. Therefore, the present study evaluates the perceived safety climate in the power industry using NOSACQ-50, which can be used to improve the overall safety culture and enhance organizational success in Pakistan’s power sector. Infact, previous research has found a link between a positive and strong safety climate and safe behavior in various industrial sectors [7]. Based on the above discussion, we propose the following research question.

Is there any statistically significant difference in the level of safety climate between organizations operating under two different employers (i.e., multinational, and local employers)?

Several multinational corporations have activities across multiple countries and continents, which includes the management of sites in both developing and developed countries [18]. In these contexts, there are differences in the safety climate perception levels among workers from different countries. For instance, a study found that Chinese workers had a higher perception of the safety climate than Vietnamese workers [19]. Bahrami et al. [20] revealed the scarcity of data on safety climate in many national contexts, Çakıt et al. [21] proposed that future research should be conducted to investigate the differences in subcultures within similar high-risk industries. Therefore, this research adds to our understanding of safety climate and contributes a comparative analysis of international employers in view of the safety climate within and outside the power sector in Pakistan. Comparative studies are necessary to comprehend how the definitions of “safety climate” may vary depending on cultural and national circumstances [22]. Based on the little evidence available in the literature, prior studies have not assessed the safety climate in the power sector from an operations and maintenance (O & M) perspective, particularly in the context of comparing multinational companies (MNCs) and local companies (LC). Thus, a theoretical gap exists here, though the power sector is an extremely hazardous working environment due to the nature of its industrial tasks. According to the, this study has been designed to reduce the knowledge gaps of SC in the power sector, especially in terms of cross-cultural employers, through the application of NOSACQ-50. The findings of the study are expected to paint a true picture of safety issues and safety performance. Our findings will add to the existing research on the safety climate in the Pakistan power sector and the surrounding geographic region and will increase awareness in this field. Furthermore, professionals can benefit from this research outcome and improve their power generation sites by considering and evaluating the identified safety climate determinants on a regular basis.

### 1.1 Study objectives

The current study aims to assess the safety climate in the power sector operated by multinational and local employers based on the perceptions of workers and leaders in Pakistan. Therefore, the objectives of the study are as follows:

- To compare the level of the safety climate in power divisions operated by two different employers’ (multinational and local) based on different determinants
- To evaluate the level of the safety climate in Pakistan compared to the NOSACQ-50 threshold.

### 1.2 Study hypothesis

This study’s hypotheses are based on several safety-relevant variables.

The hypothesis (*H*_*1*_) suggests a significant impact created by multinational employers on the safety climate in the power sector.

*H*_*1*_: Cross -cultural employers have significant influences on the overall safety climate of an organization.
*H*_*0*_: Cross-cultural employers have no significant influence on the overall safety climate of an organization.

There is a need to explore the cultural differences between workers and leaders, as these affect employee safety performance in high-risk domains, Similarly, special attention ought to be paid to how safety management and supervisory behaviors are interpreted [18]. This study hypothesizes that MNCs have a significant influence on the overall safety climate due to strong policies, procedures, management systems, and management commitment.

## 2.0 Material and methodology

### 2.1 Study area and population

The current study was carried out at power plants in Pakistan’s Punjab region. The study area was chosen on the basis that power plant contracts for operations and maintenance (O & M) were awarded to renowned multinational corporations (MNCs) and national or local corporations (LC) in this region. For this study, organizations that generate electricity using solid, oil, and gas as fuel were considered. These companies cannot be named due to confidentiality. The questionnaires were distributed to professionals (leaders and workers) across five independent power plants (IPPs) in the study area, including two O & M IPPs with a local employer and three O & M IPPs with a multinational employer. A total of 500 questionnaires were distributed (200 for two IPPs, O & M with a local employer and 200 for a third). The response rate of MNCs and LCs was 52% (n = 155), and 42% (n = 83), respectively. The total response rate for the questionnaires was 48%, which was below our expectation (i.e., we expected a response rate of more than 50%.) Similar findings were reported by Cooper & Phillips [23] who distributed 187 questionnaires to employees of the packaging production plant and had a response rate of: only merely 35%. Evans et al. [24] also reported a 31% response rate, while DeJoy et al. [25] reported 44%. To avoid any bias and to ensure privacy and anonymity, the questionnaires were filled out simultaneously by both workers and managers in all research areas.

### 2.2 Survey design

In accordance with Moda et al. [14] previous research, this study, used a cross-sectional design with a convenient snowball sampling technique to reach out to the target participants’ to assess the safety climate among professionals in the power sector. Moda et al. [14] used the same technique to collect data Snowball sampling is a non-probability sampling method [26]; that is best suited to small, difficult to reach populations. This sampling method entails primary data sources that recommend additional potential primary data sources for use in the research. More specifically, the snowball sampling method is based on initial subject referrals to generate additional subjects [27]. The participants were chosen through purposeful sampling by having contact with various professionals in the power sector and workplace units. Following This, a descriptive analysis was conducted to determine and analyze leaders’ and workers’ perceptions of the power sector’s safety climate. A quantitative method was used in the study to collect, analyze, and interpret data. Technically, the information was collected via a questionnaire. The dimension of safety climate and item questionnaire developed by Kines [28] was used in the English language. The questionnaire was not altered in any way. Nordic occupational safety researchers (tool owners) provided formal approval for the use of their developed questionnaire for this research. The questionnaire was put into Google Form to ensure easy, anonymous, and quick access. A purpose and ethics statement were also included at the top of the questionnaire to inform all participants about the purpose of the study, while also informing them that the data would be collected voluntarily, anonymously, and kept in confidence. The Advance Study and Research Board, Doctoral Program Coordination Committee, University of the Punjab, Pakistan (Ref. no. D2295/Acad) approved the study. The study involved volunteers and anonymous participants, and individual results were only made available to researchers from universities involved in the project.

The safety climate questionnaire is an important measurement tool; Zohar developed the first safety climate questionnaire, which was based on the characteristics of the manufacturing industry and included forty items that have since been widely used in subsequent studies. However, different safety climate dimensions result in differences in the variables for measurement questionnaires [29]. The currently available safety climate questionnaire is intended for use in the manufacturing, construction, energy, aviation, and health care industries. In this study, the Nordic Occupational Safety Climate Questionnaire (NOSACQ-50) questionnaire was chosen as it is one metric used to quantify specific areas or dimensions of workplace safety climate [6] and is most frequently used to assess the safety climate. NOSACQ-50 was originally developed by Nordic occupational safety researchers and presented by Kines [28] as an outcome of research actions involving a Nordic network of occupational safety researchers, and it is freely available online. The reliability and validity of the NOSACQ 50 was tested in a number of studies in a range of contexts, confirming its effectiveness as a diagnostic tool to explore the safety climate within an organization [2] in five countries (using native languages). Several research studies have validated the NOSACQ-50 across a wide range of occupational cohorts, countries, and languages [6, 30]. Previous research [6, 31–35] used and validated their results in various contexts, confirming the efficacy of the diagnostic tool to study the safety climate within organizations [2]. A design criterion used in the NOSACQ-50 development was a combination of items directly or indirectly (i.e. reversed) used to evaluate the safety climate. Higher scores are associated with a higher level of safety in positively phrased questions, such as "Management prioritizes safety over production." Lower scores correspond to a higher level of safety climate in negatively expressed statements, such as "Management always blames employees for accidents."The goal is to reduce the number of stereotyped responses, including reversed items. The reversed items are supposed to act as “cognitive speed bumps” [36] that slow down the respondent’s ability to read the text thoroughly [20]. **Table 1** shows the structure of the questionnaire, in which the first three dimensions (Dim) are related to the safety climate at the leadership level, and the last four dimensions are related to the safety climate at the level of employee.

**Table 1:** NOSACQ-50 structure.

Number	Dimensions	Total items
**Dim 1:**	Management safety priority, commitment, and competence	9
**Dim 2:**	Management safety empowerment	7
**Dim 3:**	Management safety justice	6
**Dim 4:**	Workers’ safety commitment,	6
**Dim 5:**	Workers safety priority and risk non-acceptance’,	7
**Dim 6:**	Trust in co-worker’s safety competence	8
**Dim 7:**	Trust in Efficiency of Safety Systems	7

Regarding the assessment criteria, item responses were recorded on a 4-point Likert scale, (where 4 is strongly agree, and 1 is strongly disagree). The assessment of each aspect of the questionnaire is based on the criteria summarized in **Table 2**, an easy-to-use reference for the interpretation of questionnaire results, as suggested by the NOSACQ-50 website [37] (which is managed by the Division of Safety Research of the National Research Center of the Working Environment of Denmark).

**Table 2:** Criteria for interpreting the Nordic Safety Climate Questionnaire (NOSACQ-50) results [37].

Score/ Result range	Level	Interpretation
**S>3.30**	Good	Maintain and continuing development of the SC dimensions
**3.00<s<3.30**	Fairly good	The SC dimension Slight need of improvement
**2.70<s<2.90**	Fairly Low	The SC dimension needs an improvement
**S<2.70**	Low	The SC dimension needs a great improvement

## 3.0 Results and data analysis

The data was analyzed using IBM SPSS 20.0 software.

Cronbach’s alpha was calculated to measure the reliability for sets of latent variables in the used questionnaire, and the overall alpha value for NOSACQ-50 was 0.877, indicating a satisfactory level of reliability because the value exceeded the threshold of > 0.70 [11]. **Table 3** presents descriptive statistics delineating mean and standard deviation values that compare the levels of safety climate between local and multinational employers’ samples by presenting the mean score for each item across the seven dimensions.

**Table 3:** Overall safety climate comparison between local and multinational employers.

Dimensions	Local Operated Plants (N = 83)		Multinational Operated Plants (N = 155)		
	Mean	Std. Deviation	Mean	Std. Deviation	*p-* Value
Management Safety Priority and Ability	3.10	.347 [.038]	3.19	.322 [.025]	.079
Management Safety Empowerment	3.00	.265 [.029]	3.15	.331 [.26]	.000
Management Safety Justice	3.00	.396 [.043]	3.12	.388 [.031]	.089
Workers Safety Commitment	3.11	.301 [.033]	3.21	.358 [.028]	.017
Workers Safety Priority and Risk Non-Acceptance	3.00	.428 [.047]	2.95	.433 [.034]	.345
Trust in Coworkers Safety Competence	3.12	.302 [.033]	3.23	.337 [.027]	.011
Workers Trust in Efficacy of Safety Systems	3.30	.400 [.043]	3.36	.38 [.031]	.271

[] donated Std. Error Mean, *p-*value is 0.05

**Fig 1:**
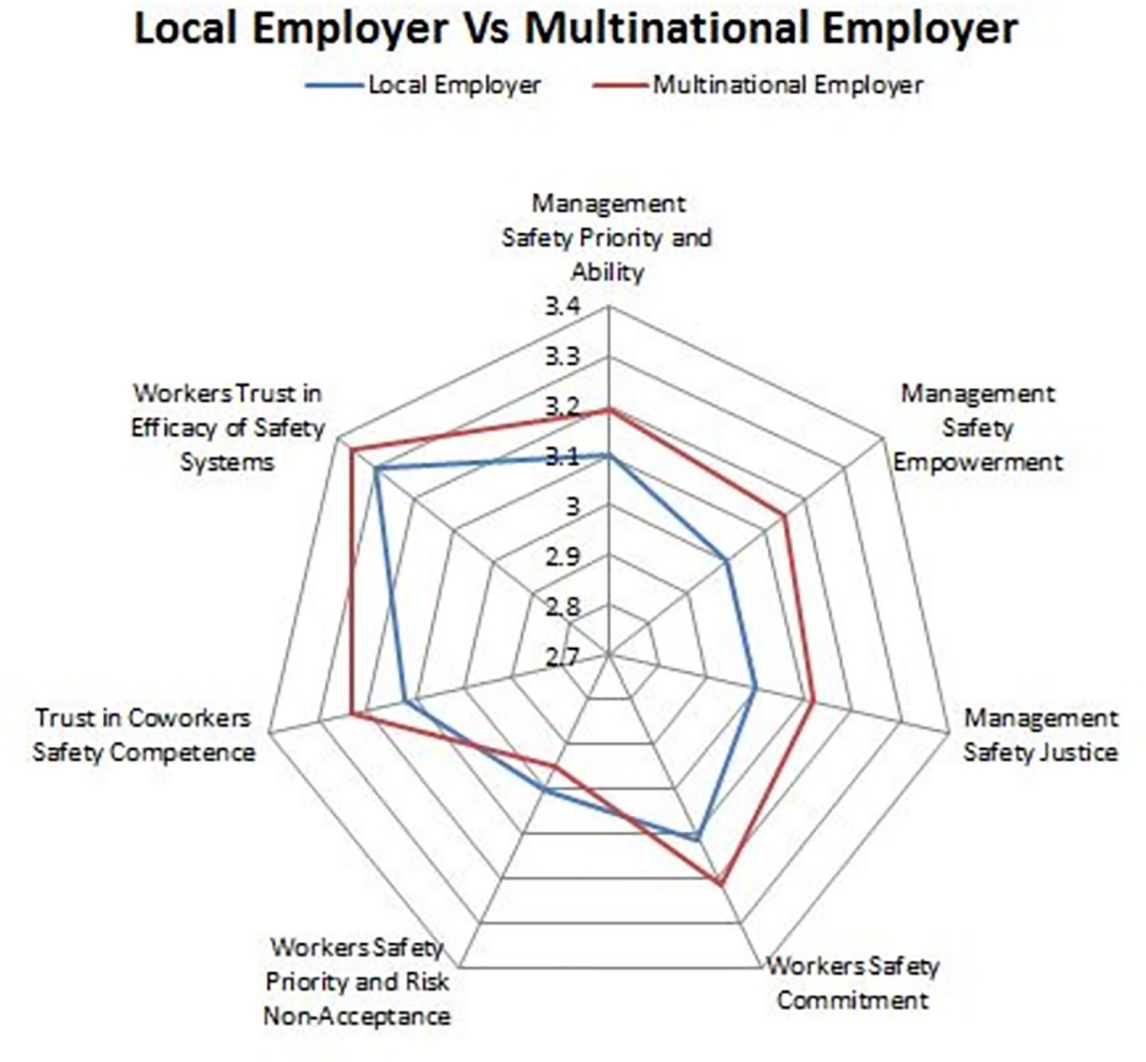
Overall safety climate scores were obtained by both employers.

A radar chart (Fig 1) was used to plot and compare mean values were compared with criteria summarized in Table 2, which provides a reference for the interpretation of the NOSACQ-50 questionnaire results.

Further, to compare leaders’ and workers’ perceptions of safety climate in two different employer settings, an independent *t-test* (two-tailed) was performed on the mean difference between the two populations to determine whether the difference between the mean scores of samples was statistically significant or not, as shown in Table 4. This parametric test is trustworthy and widely used to compare the means of two samples across similar variables.

**Table 4:** Safety climate comparison between local & multinational employers leaders & workers perception.

	Leaders Perception					Workers Perception				
Dimensions	Local Operated Plants (N = 42)		Multinational Operated Plants (N = 69)			Local Operated Plants (N = 41)		Multinational Operated Plants (N = 86)		
	Mean	S.D	Mean	S.D	*p-* Value	Mean	S.D	Mean	S.D	*p-* Value
**Management Safety Priority and Ability**	3.15	.292	3.17	.323	.484	3.06	.394	3.18	.324	.086
**Management Safety Empowerment**	3.04	.225	3.20	.348	.004	2.96	.299	3.11	.313	.010
**Management Safety Justice**	3.03	.298	3.19	.44	.029	3.03	.480	3.07	.334	.623
**Workers Safety Commitment**	3.13	.298	3.20	.389	.274	3.09	.305	3.22	.333	.024
**Workers Safety Priority and Risk Non-Acceptance**	3.04	.389	3.15	.432	.148	2.96	.468	2.78	.357	.029
**Trust in Coworkers Safety Competence**	3.10	.315	3.24	.360	.030	3.14	.289	3.22	.319	.169
**Workers Trust in Efficacy of Safety Systems**	3.28	.376	3.34	.415	.395	3.32	.426	3.37	.365	.524

*p* value is 0.05

## 4.0 Discussion

The findings show that MNCs had a higher response rate than LCs because workers and managers in locally employed companies are less aware of the safety climate and its benefits. Another reason based on our observations during collection of data is that employees at local companies have more job insecurity and fear of retaliation than their counterparts at MNCs. This clearly demonstrates the cultural differences between multinational and local employers.

Table 3 displays the overall safety climate mean, SD, and *p-value* for MNCs and LC. Furthermore, an *independent t-test* was applied to examine the perception of leaders and workers in both setups highlight where actual differences exist. An *independent t-test* revealed that there is a significant difference in three dimensions; one related to management, i.e., Management Safety Empowerment, dim 2, and the other two pertaining to workers, i.e., Workers’ Safety Commitment Dim 4 and Trust in Coworkers’ Safety Competence (Dim 6). According to the results of these tests, the safety climate scores of multinational employers are generally higher than those of local employers. The overall safety climate is depicted in (Fig 1), which compare the findings to the thresholds prescribed by the NOSACQ50. This comparison clearly shows higher mean scores in each dimension for leaders in MNCs compared to the NOSACQ50 threshold.

The presented results for management safety commitment revealed no significant differences between the studied groups. In practice, the perception of management’s safety commitment and priority is one of the most commonly assessed factors in the safety climate theme [38] when determining safety performance [39]. Small businesses, on the other hand, lack both the financial resources and the managerial commitment to improve their safety performance compared to large companies [39]. This finding aligns with the outcomes that MNC managers are perceived to adequately demonstrate safety commitment compared to LC management, although the difference was not found to be significant. Based on the responses of employees from MNCs, management encourages employees to ensure safety even when production is compromised.

With regards to the differences observed between groups related to leaders’ safety empowerment, the results confirm that MNCs’ employees believe that management trusts their workers and conveys this trust by empowering them and valuing their contributions. However, LCs’ management (*M = 3*.*04*) and workers’ accumulative results (*M = 2*.*96*) show that an essential element of safety empowerment requires improvement. According to Kines [28] managers should improve their belief in employees’ ability to competently participate in safety decisions and in dealing with safety. Management commitment and empowerment are directly related to workers’ safety commitment; if management only blames the workers instead of empowering them, the overall Perceived Organizational Support (POS) will be impacted. POS is based on the presumption that “employees in an organization form global beliefs concerning the extent to which the organization values their contributions and cares about their well-being”, and that such beliefs increase employees’ emotional commitment to the organization [28]. Our study findings support this relationship in that a significant difference was observed in workers’ perception of management safety empowerment and Worker’s safety commitment employed across both employers.

When researching the Management Safety Justice’s (Dim 3) safety climate factor, a non-significant difference was observed in the overall responses in both setups. Further investigation revealed that workers and leaders appointed by LCs share the same perception of management safety justice; this dimension includes questions related to blaming employees, conducting fair investigations, looking for causes, and listening to staff involved in an accident. When comparing both setups, leaders’ perceptions differed, but workers’ thoughts were the same. Based on our cultural observation this could be because employees seldom complain, as it is deemed culturally unacceptable to question a figure of authority. However, managers may not feel the need to consult workers because they are less educated and many of them are informal workers. This highlights management’s reactive approach towards safety. According to Daniel et al. [40] blame may be a barrier to learning, when accountability and criticism are dominant features of the work situation. Safety tends to be excessively managed through formal procedures as a means of self-preservation, thereby resulting in an increasingly prescriptive and inflexible compliance culture.

According to existing research, organizational commitments are negatively related to employee withdrawal behaviors, including absenteeism, withdrawal cognitions, turnover, and turnover intentions; in contrast, they are positively associated with job performance, and organizational citizenship behavior [25] with unrealistic targets and a lack of appreciation of safety performance create work environments that impede safety implementation. Our study shows a significant difference among workers’ perceptions of safety commitment in MNCs and LCs. Martin [26] had similar findings in China and Indonesia, where it was found that LC employees tended to follow instructions regardless of safety policy, and assumed that they had no impact on safety performance in their workplace.

**Fig 2:**
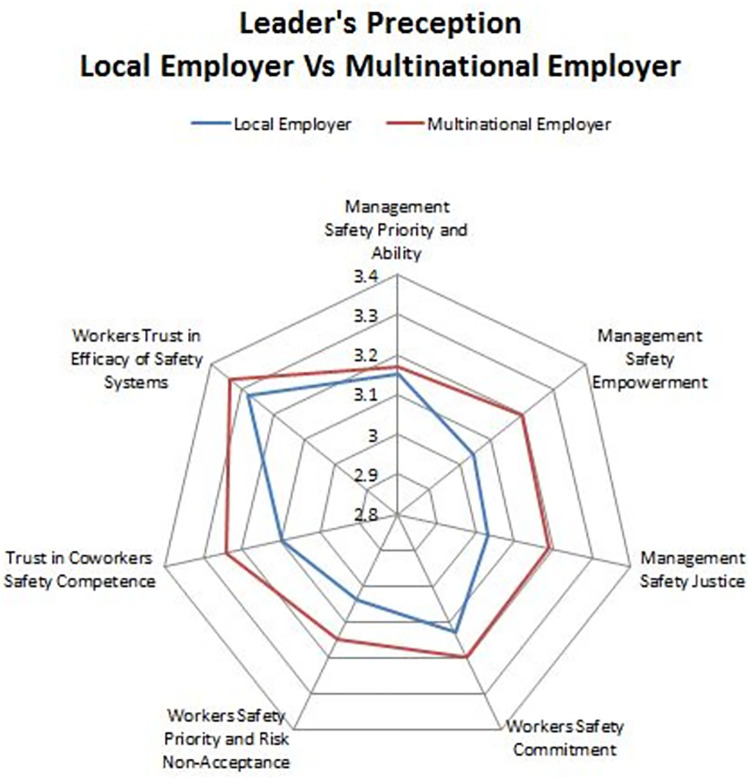
Leader’s perception on safety climate in both MNCs & LC.

**Fig 3:**
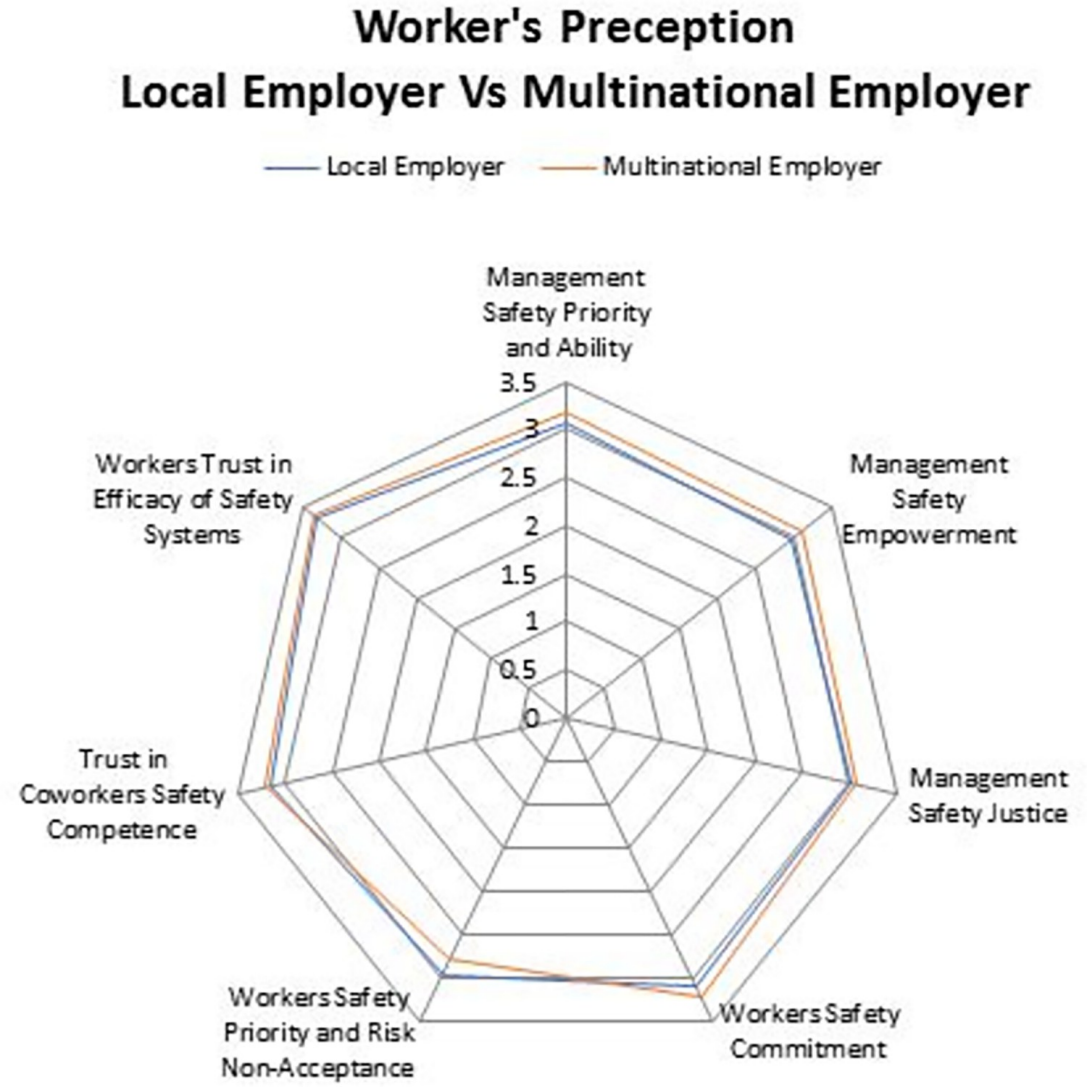
Worker’s perception on safety climate in both MNCs & LC.

Workers’ Safety Priority and Risk Non-Acceptance (Dim 5) (LC: M = 3.00, SD = 0.428; MNC: M = 2.95, SD = 0.433, *p =* 0.345). Our study results are supported the findings of a study conducted in Iran, as well as another in Hong Kong, which showed that the importance of co-workers in the SC of the construction industry is more important than in developed countries [40]. In this regard, our own findings revealed a significant difference in the mean scores between workers employed by LCs and MNCs. Fig 2, shows stronger perception among leaders in MNCs. Whereas, in (Fig 3) workers had higher mean scores. This is the only dimension where the mean score in MNC was found to be lower than LC. A more in-depth exploration reveals that MNC employees display more confidence and are willing to take more risks at work. Furthermore, unreasonable work targets and a failure to recognize safety performance creates work environments that impede safety implementation. This finding is consistent with the results of Zhang et al. [41] who found that variance and change in construction projects induce changes in relative priorities and production pressure, thereby causing a shift in safety performance. Future, Gillen et.al. study also found that both union and nonunion workers considered their jobs to be overwhelmingly satisfying, despite the fact that they had all recently been injured [42].

Workers’ trust in the efficacy of safety systems (Dim 7) was ranked the highest in both setups, which includes training, clear-cut goals, safety inspections, resources, and planning. Our results show that employees’ overall perception was positive when it comes to trust in the efficacy of safety systems. This may be because the overall safety system in Pakistan is not strong, legislation is quite weak, and implementation of the existing legislative framework is still a big challenge; power plants are relatively automated and workers in both setups are satisfied with the adopted safety measures. Another reason is that workers are typically hired through contractors, who are mostly locals, in both sets-up. Here, results from Daniel [40] could be used to demonstrate that workers from religious backgrounds are more interested in positive safety perceptions than those from non-religious backgrounds. Moreover, compared to employees that are directly employed by the main contractor, workers employed by subcontractors tend to have a more positive perception of safety.

Compared to other dimensions, dim 6, which relates to trust in coworkers’ safety competence produced better results in employees from LCs. Seo et al. [43] identified co-worker safety competence as one of the five dimensions of safety climate. However, the complexities of trust must also be considered. According to Conchie and Donald [44], if coworkers are trusted blindly, double-checking safety-critical tasks may be overlooked, and mistakes may go undetected. This study’s results support this t in the case of LC workers. If all other dimensions were ranked lower on the chart, this perception must support a blind trust strategy in order to safely complete the work without considering safety.

Interestingly, non-significant low scores were observed in both setups related to worker safety priority and risk non-acceptance. When delineating the results of most individual reverse questions, MNC employees usually do not undertake unsafe or risky behaviors. However, when it comes to meeting deadlines or working in other stressful situations, employees have a tendency to disregard safety. This finding is consistent with that of Loosemore et al. [26] who discovered that unrealistic and uncomfortable work targets, as well as a lack of recognition of safety performance, create work environments that impede safety implementation. This finding aligns with that of Zhang et al. [41] who also found that variability and change in construction projects cause fluctuations in safety performance due to shifting priorities and production pressures. Similar conditions exist in Indonesia, where insufficient safety resources and job pressure cause safety to become less of a priority.

This study’s results are also aligned with Gittleman et al. [45] work which found that safety climate observation scores vary between two distinct subcultures in the nuclear industry. Previous research has found that a safety climate is strongly related to safety participation and, in order to foster a safety climate in any organization, safety training, safety communication, and safety systems should be actively encouraged within the establishment [46–48]. MNCs’ overall safety climate meets the NOSACQ threshold as they primarily adhere to national and international safety standards. Safety regulations. These results show that respondents from LCs scored higher on management-related dimensions than on worker-related dimensions. This draws our attention to the on-the-ground safety culture and radar chart, which shows similar responses from workers in both setups, albeit different s in responses from management/leaders. This demonstrates that LC employees are hesitant to criticize management for fear of retaliation. Despite the fact that the survey results were kept anonymous, significant gaps were found in the responses. In the case of MNCs, however, distribution is smooth across all dimensions. Griffin and Curcuruto [18] hypothesized that the relationship between perceived managerial commitment to safety and compliance with risk-taking behaviors varies across cultures. Consequently, strengthening the management-related dimensions that demonstrate the highest mediation relations and overall safety climate is of critical importance, since leadership is considered a precedent for safety climate. Through supervision and communication, employees begin to understand "what is important” in their workplace. Employees’ perception and prioritization of safety will improve if administrators communicate the significance of safety behaviors, practices, and procedures [49].

Several implications emerge based on the findings of this study. First, the study itself improved leaders’ & workers’ perceptions of the safety climate in Pakistan’s power sector. Furthermore, the findings of this study contribute to an understanding of the possible range of key elements, such as management safety engagement, across national contexts. Second, it provides insights into how national systems (such as legislative and cultural systems) can create values that effectively moderate the link between safety climate and outcomes. Furthermore, improved communication and interaction will lead to positive collaboration and, ultimately, increased trust in the safety system, which will likely improve the safety climate and productivity. Finally, inculcating the safety climate concepts at each level will help this sector become more proactive rather than reactive when it comes to safety measures.

A key strength of the present study is the capability to adapt the validated NOSACQ-50 questionnaire to measure safety climate in various contexts. Additionally, this is the first study to compare cross-cultural intrusion in MNCs and local establishments in the power sector in Pakistan. Due to the limitations of the study’s geographical area and research objects, the current findings have limited generalizability. However, the study’s approach can be easily adjusted and applied to other areas, including power sectors in various contexts.

## 5.0 Conclusions

This paper was aimed of this paper was to address the lack of available cross-cultural employer assessments of the safety climate in the power sector. To this end, a comparative analysis was conducted involving multinational and local employers of a power plant based in Pakistan. The outcomes offer some new and surprising similarities and differences in the safety climates across different employers; Based on the collected responses, there is a more a positive and stronger safety climate in power plants operated by MNCs compared to LCs. Management safety empowerment, worker safety commitment, and trust in coworkers’ safety competence all had significantly different scores across the categories of employers. Even in an anonymous survey, workers’ responses to management-related dimensions in LCs depict employees’ fear of retaliation. The involvement and perception of LC leaders must therefore be improved. However, the SC scores obtained by MNCs were evenly distributed across all dimensions. Our findings suggest that this is not always the case and that the concept of safety climate, can be misleading when considered in isolation as an indicator of health and safety performance. This becomes clear only when comparing different employers using a similar standardized tool; Furthermore, this raises additional practical questions for managers about the value on focusing on safety climate as a strategy for intervention. Theoretically, our findings also highlight the mediating role of organizational factors like management commitment and cultural differences in shaping the safety climate. To improve the overall safety system, improved safety communications, training, and implementation of safe work systems are required at the local level. More stringent laws and regulations are needed in Pakistan to compel management to ensure workplace safety. Higher scores among MNC respondents show the proper implementation of corporate safety rules and management commitment to providing a safe workplace. These results reveal that if management’s prioritizes safety by empowering the team, and practicing safety measures, employees will be more likely to engage in positive and sustained safety behaviors. The study concludes that promoting an efficient and positive safety climate in the power sector is a long process and that can only be accomplished if all workers and leaders take an active role in this. Pakistan has inadequate safety regulations due to insufficient government policies, will, and regulatory body monitoring. The findings of this study provide a strong basis for regulators to improve safety regulation in line with more developed countries. In future, the results obtained from the Punjab power sector can be compared of safety climate assessments in other industries and used as a baseline for power sector companies worldwide. As previously mentioned, safety climate is a leading indicator (rather than a lagging indicator of safety). The evaluation of the status and progress of the safety climate within an organization will help to identify key focus areas and workers’ perceptions about safety, which will lead to the timely establishment of risk reduction action plans for high-risk groups.

## Supporting information

S1 Data Survey data file. (XLSX)
